# Hematological Response to Particle Debris Generated During Wear–Corrosion Processes of CoCr Surfaces Modified with Graphene Oxide and Hyaluronic Acid for Joint Prostheses

**DOI:** 10.3390/nano14221815

**Published:** 2024-11-13

**Authors:** María L. Escudero, Maria C. García-Alonso, Belén Chico, Rosa M. Lozano, Luna Sánchez-López, Manuel Flores-Sáenz, Soledad Cristóbal-Aguado, Rafael Moreno-Gómez-Toledano, Soledad Aguado-Henche

**Affiliations:** 1 Centro Nacional de Investigaciones Metalúrgicas (CENIM), Consejo Superior de Investigaciones Científicas (CSIC), Avenida Gregorio del Amo, 8, 28040 Madrid, Spain; escudero@cenim.csic.es (M.L.E.); bchico@cenim.csic.es (B.C.); luna.sanchez@cenim.csic.es (L.S.-L.); 2 Centro de Investigaciones Biológicas-Margarita Salas (CIB Margarita Salas), Consejo Superior de Investigaciones Científicas (CSIC), C/Ramiro de Maeztu, 28040 Madrid, Spain; rlozano@cib.csic.es; 3 Program in Advanced Materials and Nanotechnology, Doctoral School, Universidad Autónoma de Madrid, Ciudad Universitaria de Cantoblanco, 28049 Madrid, Spain; 4 Program in Translational Medicine, Doctoral School, Universidad de Alcalá, 28801 Alcalá de Henares, Madrid, Spain; manuel.floress@uah.es; 5 Universidad de Alcalá, Area of Human Anatomy and Embryology, Department of Surgery, Medical and Social Sciences, Campus Científico-Tecnológico, Crta. Madrid-Barcelona, Km. 33,600, 28805 Alcalá de Henares, Madrid, Spain; rafael.moreno@uah.es; 6 Universidad de Alcalá, Department of Nursery, Campus Científico-Tecnológico, Crta. Madrid-Barcelona, Km. 33,600, 28805 Alcalá de Henares, Madrid, Spain; soledad.cristobal@uah.es; 7 Principe de Asturias University Hospital (HUPA), Campus Científico-Tecnológico, Av. Principal de la Universidad, s/n, 28805 Alcalá de Henares, Madrid, Spain

**Keywords:** wear–corrosion particles, hematological analysis, graphene oxide, hyaluronic acid, CoCr, inflammatory response

## Abstract

Various surface modifications to increase the lifespan of cobalt–chromium (CoCr) joint prostheses are being studied to reduce the wear rate in bone joint applications. One recently proposed modification involves depositing graphene oxide functionalized with hyaluronic acid (a compound present in joints) on CoCr surfaces, which can act as a solid lubricant. This paper analyzes the biological alterations caused by wear–corrosion phenomena that occur in joints, both from the perspective of the worn surface (in vitro model) and the particles generated during the wear processes (in vivo model). The analysis of the inflammatory response of macrophage was performed on CoCr surfaces modified with graphene oxide and functionalized with hyaluronic acid (CoCr-GO-HA), before and after wear–corrosion processes. The wear particles released during the wear–corrosion tests of the CoCr-GO-HA/CoCr ball pair immersed in 3 g/L hyaluronic acid were intra-articularly injected into the experimental animals. The hematological analysis in vivo was made considering a murine model of intra-articular injection into the left knee in male adult Wistar rats, at increasing concentrations of the collected wear particles dispersed in 0.9% NaCl. Non-significant differences in the inflammatory response to unworn CoCr-GO-HA surfaces and control (polystyrene) were obtained. The wear–corrosion of the CoCr-GO-HA disk increased the inflammatory response at both 72 and 96 h of material exposure compared to the unworn CoCr-GO-HA surfaces, although the differences were not statistically significant. The pro-inflammatory response of the macrophages was reduced on the worn surfaces of the CoCr modified and functionalized with graphene oxide (GO) and hyaluronic acid (HA), compared to the worn surfaces of the unmodified CoCr. The hematological analysis and tissue reactions after intra-articular injection did not reveal pathological damage, with average hematological values recorded, although slight reductions in creatinine and protein within non-pathological ranges were found. Some traces of biomaterial particles in the knee at the highest concentration of injected particles were only found but without inflammatory signs. The results show the potential benefits of using graphene in intra-articular prostheses, which could improve the quality of life for numerous patients.

## 1. Introduction

CoCr alloys were widely used in the femoral heads of joint prostheses due to their high wear–corrosion performance [1]. Nevertheless, the persistent sliding motion in joint prosthesis contact areas gave rise to wear–corrosion phenomena. Continuous sliding and corrosion combined generate stuck deposits on the worn joint prosthesis surfaces. The examination of these deposits in the prostheses retrieved from cadavers has revealed layers rich in nanocrystalline graphite and amorphous sp^2^ carbon [2], with a sp^2^ bonding fraction of approximately 80% [3,4,5,6,7,8]. While these layers can serve as protective and lubricating barriers against physiological fluids, they fail to cover the entire worn surface, leading to the continuous generation of metallic debris in regions exposed to wear–corrosion. Understanding both the composition of the debris and its interactions with surrounding cells, as well as the potential for migration to other parts of the body, is crucial for comprehending its association with local and systemic toxicity [9,10,11,12,13,14,15,16].

One primary motivation for prosthetic replacement is the adverse biological reactions caused by metal debris. This unresolved challenge drives ongoing efforts to discover novel protective strategies for CoCr joint prostheses to reduce wear–corrosion rates and minimize the biological foreign body response. To enhance tribocorrosion performance and mitigate the release of metallic debris, the researchers of this study have advocated for the adoption of graphene-based chemical modifications on the surface. This approach aims to emulate the presence of carbon-enriched tribological layers identified in worn areas of in vivo-retrieved prostheses, as these layers have been linked to wear reduction [2,3,5,6,7,8].

The strong covalent bonding between atoms in the carbon network enhances the surface’s exceptional mechanical properties. Detaching these layers during tribocorrosion processes releases debris that can interact with the biological environment, potentially leading to adverse reactions in vivo.

The posterior functionalization of graphene-based compounds with biological molecules responsible for joint lubrication, such as hyaluronic acid (HA), through oxygen groups, could serve as an effective strategy to improve tribocorrosion performance without compromising biocompatibility [17].

Hyaluronic acid, the primary lubricating component of synovial fluid, is a dense polysaccharide containing hydroxyl and carboxyl groups. Its outstanding viscoelastic properties play a crucial role in lubricating articular cartilage [18]. The approach of chemically concentrating HA on the surface of graphene oxide (GO) aims to replicate the behavior of articular cartilage, offering lubrication in the joints.

In the model of induced osteoarthritis in rats by Liu et al. [19] and in rabbits developed by Wang et al. [20], the treatment with graphene oxide and hyaluronic acid does not elicit an inflammatory response or a safety-related risk, so its use in combination with traditional CoCr could improve the biological properties of the material in the context of intra-articular prostheses.

The main aim of this study is to analyze the inflammatory response of macrophages (in vitro tests) to CoCr surfaces modified with graphene oxide and hyaluronic acid, both before and after wear–corrosion tests, as well as the hematological response in rats (in vivo tests) to the wear particles generated during those tests. Different concentrations of the collected wear particles, dispersed in 0.9% NaCl, were intra-articularly injected into the left knee of rats to simulate an increasing number of particles generated in joint replacements rising over the device’s lifetime.

## 2. Materials and Methods

The animal experiment description followed the ARRIVE guidelines.

### 2.1. Wear–Corrosion Tests of Modified CoCr with Graphene Oxide and Hyaluronic Acid

#### 2.1.1. Materials and Reagents

Biomedical-grade CoCr alloy was purchased from Edge International (Dayton, OH, USA) with the nominal chemical composition (wt.%) of 27.62% Cr, 5.50% Mo, 0.8% Mn, 0.62% Si, 0.04% C, 0.32% Fe, 0.170% N, 0.10% Ni, 0.02% W, 0.001% S, and 0.003% P, and a balance of Co.

CoCr disks, measuring 38 mm in diameter and 3 mm in thickness, were abraded using SiC abrasive papers ranging from 340 to 2000 grit size, followed by successive polishing with 3 µm and 1 µm diamond paste. All the samples were rinsed with ultrapure water and then with acetone in an ultrasonic bath for 15 min. They were stored in a desiccator until use.

For the deposition of graphene oxide on CoCr and subsequent functionalization with hyaluronic acid, the following reagents were used: 3-aminopropyl-triethoxysilane (APTES) and adipic dihydrazide 98% (ADP) were purchased from Merck (Darmstadt, Germany), Sigma-Aldrich (St. Louis, MO, USA), and Thermo Fisher Scientific (Waltham, MA, USA), Acros Organics (Geel, Belgium), respectively. The graphene oxide (GO) was provided as an aqueous suspension with a concentration of 4 g/L by Grupo Antolin Holding (Burgos, Spain). Hyaluronic acid (HA) sodium salt from Streptococcus equi was supplied by Sigma-Aldrich Chemie GmbH, St. Louis, MO, USA.

#### 2.1.2. Deposition of Graphene Oxide Functionalized with Hyaluronic Acid on CoCr Disks (CoCr-GO-HA Surface)

Briefly, CoCr disks were immersed in 5M NaOH for two hours for surface hydroxylation. After that, activated surfaces were immersed at room temperature for 1 min in a pre-hydrolyzed APTES solution (2 vol % in isopropanol-water (200:1 *v*/*v*) and stirred for 1 h). Then, silane-coated samples were curated at 45 °C for 24 h. The next step was incubating silane-coated samples in 4 g/L graphene oxide aqueous suspension at 60 °C for 24 h. At this point, graphene oxide deposits were synthesized on the CoCr surfaces (hereafter named CoCr-GO).

Functionalization of the CoCr-GO surface with HA (CoCr-GO-HA) was made using an intermediate agent, adipic dihydrazide 98% (ADP). CoCr-GO disks were immersed in an aqueous solution consisting of N-hydroxysuccinimide (NHS) at a concentration of 15 g/L and 1-(3-(dimethylamino) propyl)-3-ethyl-carbodiimide hydrochloride (EDC) at a concentration of 5 g/L for 24 h at temperature of 25 °C, to activate the carboxylic groups of the GO-coated surface for further reaction. Subsequently, ADP anchoring on the activated CoCr-GO disks was performed via immersion in an aqueous ADP solution of 10.4 g/L concentration for 24 h at 25 °C. Then, all the samples were rinsed with ultrapure water and immersed in a phosphate buffer solution (PBS) containing HA (1 g/L), NHS (7.5 g/L) and EDC (2.5 g/L) for 24 h and 25 °C. This solution was previously under magnetic stirring for 24 h to activate the –COOH groups of HA. The authors previously published the procedure for obtaining the CoCr-GO and CoCr-GO-HA multilayer systems in [21,22], respectively.

#### 2.1.3. Debris Collected from Wear–Corrosion Process

Wear–corrosion tests of CoCr-GO surfaces functionalized with HA (CoCr-GO-HA; hereafter biofunctionalized CoCr) versus CoCr balls of 8 mm diameter as pins were carried out in a MT/30/NI tribometer (Microtest, Madrid, Spain) with pin-on-disk configuration.

The wear test parameters are summarized as follows: the load applied was 5 N at a 120 rpm rotational rate for a sliding distance of 30,000 m (682,000 cycles). The CoCr ball on the CoCr-GO-HA disk was immersed in an aqueous solution containing HA at a physiological concentration typical of healthy joints (3 g/L), which is the primary component of synovial fluid known for its lubricating properties. This solution was continuously recirculated throughout the test, simulating sliding distances equivalent to approximately one year of in vivo service [23].

After wear–corrosion tests, the worn biofunctionalized CoCr surfaces were submitted for the study of the macrophage response. On the other hand, the collected wear particles were dispersed in 0.9% NaCl at increasing concentrations: 0.63 mg/mL, 1.65 mg/mL, 1.83 mg/mL, and 2.35 mg/mL. An increase in concentration was selected to simulate the fact that the number of particles generated in joint replacements will rise over the device’s lifetime; that is, the particle concentrations found will vary based on each patient’s device lifespan.

Then, the wear particles were delivered for intra-articular injection in the experimental animals.

Twelve wear–corrosion tests were conducted with the biofunctionalized CoCr surfaces.

#### 2.1.4. Chemical Composition and Size Distribution of Wear Particles

The wear particles released in the wear–corrosion tests were collected and extracted from the hyaluronic acid solution by centrifugation at 14,513× *g* for 3 h. Once the supernatant was discarded, the particles were washed and filtered with plenty of water. Durapore membrane filters, with a diameter of 47 mm and a pore size of 0.10 µm, were used. The mass of the collected particles was calculated by weighing the filters before and after filtration. Microbalance was used to give the necessary sensitivity. In total, four centrifugations were performed to obtain increasing wear particle concentrations, and the particle mass from each isolation was dispersed in 0.9% NaCl before being sent for in vivo tests.

MasterSizer 3000 (Malvern Panalytical, Malvern, UK) with laser diffraction technology has been used to measure particle size distribution from 10 nm to 3.5 mm. It is equipped with a red He-Ne laser at 632.8 nm, a blue LED laser at 470 nm, and a Hydro SV small volume dispersion unit with stirring.

Inductively Coupled Plasma Optical Emission Spectroscopy (ICP-OES, Agilent Technologies DVD5100, Santa Clara, CA, USA) performed a semi-quantitative analysis of the particle dispersion. The collected wear particles were dispersed in a 0.9% NaCl solution. Measurements were carried out using reference standards for the following analyzed elements: Co, Cr, and Si.

### 2.2. In Vitro Tests: Macrophages Cell Cultures

A mouse macrophage J774A.1 cell line was chosen to analyze the inflammatory response of biofunctionalized CoCr surfaces before and after wear–corrosion tests. The cell line was purchased from the DSMZ Human and Animal Cell Bank (DSMZ, Braunschweig, Germany).

Before the cell culture assay, metallic disks were sterilized under UV in an active vertical flux cabin for 5 min on each side. After sterilization, the disks were placed into a sterile tissue culture dish, and cells were seeded onto them.

The macrophages were cultured in Dulbecco’s Modified Eagle Medium (DMEM 41966; Gibco ThermoFisher Scientific, Waltham, MA, USA) supplemented with 10% heat-inactivated fetal bovine serum (FBS; Gibco ThermoFisher Scientific, Waltham, MA, USA) and with a mixture of antibiotics (penicillin at 100 units/mL and streptomycin at 100 µg/mL, Gibco, BRL), named hereafter as “complete cell culture medium”.

Before cell culture, unworn biofunctionalized disks were incubated for four days in 3 g/L HA, the same medium used in the wear–corrosion tests. In the biofunctionalized CoCr surfaces submitted to wear–corrosion, the worn disk surface was facing up for direct contact with cells.

The cells were seeded at 12,500 cells/cm^2^ cell density for 72 h and 10,000 cells/cm^2^ for 96 h in 7 mL of complete cell culture medium at 310.15 K and 5% CO_2_.

Experiments were conducted as three independent assays.

#### Inflammatory Response of J774A.1 Macrophages to Biofunctionalized CoCr Surfaces

After macrophage culture, the cell medium was collected and centrifuged (5 min at 1024× *g*). The quantitative determination of cytokines in the cell medium was measured with commercial kits specifically designed for the detection of tumor necrosis factor-α (TNF) as a cytokine indicator of a pro-inflammatory response (murine TNF-α Elisa Kit, Diaclone, Besançon, France) and interleukin IL-10 as a cytokine indicator of an anti-inflammatory response (murine IL-10 Elisa Kit, Diaclone, Besançon, France).

The inflammatory responses to unworn and worn materials were analyzed by the ratio TNF-α/IL-10.

Protein concentration was measured in the cell extracts as described in [24] and used here to normalize cytokine concentrations.

The TNF-α/IL-10 ratios were the average of three independent assays in triplicate.

### 2.3. In Vivo Tests: Intra-Articular Injection of Particles in Wistar Rats

#### 2.3.1. Animals

The in vivo response to wear particles was studied in male adult Wistar rats of approximately 250 ± 10 g body weight and four months. The rats were given a seven-day acclimation period at the Animal Experimentation Center of the University of Alcalá de Henares (Madrid) before the experiments began. Madrid’s Ethics Committee for Regional Clinical Research (CEIC-R) reviewed and approved the protocol. The animals were treated following the University Ethics Committee, Spanish regulations (RD 53/2013), and the European Union’s guidelines for treating animals used for experimental purposes (86/609CEE; recommendation 2007/526/CE).

The rats were housed in polycarbonate cages (n = 4/cage) and maintained in a controlled environment with a 12 h-dark/12 h-light cycle at 295.35 K and relative humidity of 50–70%. Pellets, nutritionally balanced food, and water were provided ad libitum. Following three days of regular feeding, the animal weight and feed intake were measured and recorded daily at 9:00 a.m.

#### 2.3.2. In Vivo Experimental Design

The particles were intra-articularly injected into the left knee of each animal using a special Hamilton syringe (100 µL model 710LT SYR) and a 27G × 1/2″ needle (0.4 × 12 mm) from the NIPRO brand. The knee joint was chosen, as it best combines joint size and accessibility. Following anatomical considerations and employing an aseptic technique (disinfection of the area with 96° alcohol), adverse effects were avoided. The rats were previously anesthetized with 5% isoflurane (short-duration anesthesia to allow for animal immobilization) at a flow rate of 1 L O_2_ per minute, and the anatomical area was shaved. While under anesthesia, they remained at a concentration of 2% isoflurane throughout the inoculation procedure. Considering the rat’s knee anatomy, the left knee was flexed, and the anterior tibial tuberosity and patellar tendon were identified. Using an explorer’s nail, the interarticular line was located, and access was gained above the tibial plateau and along the medial edge of the patellar tendon (anteromedial access), guiding the needle vertically upward. One month after injection, blood extraction was performed prior to euthanasia in a CO_2_ chamber, followed by the retrieval of the injected limb.

The rats were divided into seven different groups. All of them were injected with a volume of 30 µL. The control group (0) was injected with a 0.9% NaCl solution. Group (1) was injected with hyaluronic acid at 10 mg/mL. Group (2) received a suspension of commercial reduced graphene oxide (from Sigma Aldrich) at 1 mg/mL concentration in 0.9% NaCl. The particles from the wear–corrosion tests were dispersed in a 0.9% NaCl solution in the following concentrations: an injection of wear particle concentration at 0.63 mg/mL in group (3); an injection of wear particle concentration at 1.65 mg/mL in group (4); an injection of wear particle concentration at 1.83 mg/mL in group (5); an injection of wear particle concentration at 2.35 mg/mL in group (6). The experimental design used is summarized in Table 1.

#### 2.3.3. Histological Analysis

After sacrificing the animal (at the end of the one-month trial), the left knee was obtained and placed in buffered formalin at pH 7 to prevent sample decalcification. It underwent histological processing for hard tissues using the EXAKT micro-grinding system (EXAKT Advanced Technologies GmbH, Norderstedt, Germany) following the phases of fixation, embedding, sectioning–polishing, and staining. To study the joint cavity, the stains used were hematoxylin–eosin, toluidine blue, and Masson’s trichrome [25].

#### 2.3.4. Blood Analysis

After 30 days of inoculation, and prior to euthanizing the animal in a CO_2_ chamber, 1.5 mL of intracardiac blood was extracted for analysis. The serum samples were prepared through centrifugation at 3000× *g* for 15 min. The serum was then separated and transferred into 1.5 mL Eppendorf tubes, which were stored in a cold chamber for transport to the laboratory. Hematological analysis was conducted by UNILABS (Madrid, Spain), using a Sysmex Kx-21n hematology autoanalyzer (Sysmex Corporation, Kobe, Japan) to assess the hematological parameters.

The hematological analysis encompassed both red and white blood cells, as well as biochemical parameters. The whole-blood hematological parameters measured included hemoglobin (Hgb), hematocrit (Hct), white blood cell (WBC) count, red blood cell (RBC) count, mean corpuscular volume (MCV), mean corpuscular hemoglobin concentration (MCHC), and platelet (PLT) count.

The biochemical parameters analyzed in the serum samples were creatinine (Crea), aspartate aminotransferase (AST), alanine aminotransferase (ALT), lactate dehydrogenase (LDH), total protein (TP), triglyceride (TG), and cholesterol (CHO).

The coagulation study evaluated the prothrombin time (PT), thromboplastin time (TT), and fibrinogen (F). The experimental methodology was followed as described in [26].

#### 2.3.5. Statistical Treatment

The comparative analysis of the quantitative variables was first conducted using Pearson correlation. Subsequently, the data distribution was analyzed using the D’Agostino–Pearson and Shapiro–Wilk normality tests. Subsequently, a one-way ANOVA or Kruskal–Wallis followed by a Bonferroni or Dunns’ test, respectively, were carried out. The *p*-values presented in the figures and tables corresponded to the post hoc test. All the statistical analyses were performed using the GraphPad Prism 7.0 software (GraphPad Software Inc., San Diego, CA, USA), IBM SPSS Statistics for Windows software, version 27 (IBM Corp., Armonk, NY, USA), and STATGRAPHICS^®^ Plus version 5.1 (Statistical Graphics Corp. Warrenton, VA, USA). *p* < 0.05 was considered statistically significant.

## 3. Results and Discussion

### 3.1. Inflammatory Response to Unworn and Worn Biofuncionalized CoCr Surfaces

The toxicity and issues arising from the products generated in the wear–corrosion processes of the Me/Me pair are well known. These include particulate wear debris, free metallic ions, and inorganic metal salts or oxides [27].

Several reports [28,29,30,31,32,33] have documented patients with MoM implants exhibiting systemic symptoms, including neurological disorders, cardiomyopathy, and hypothyroidism. All the patients had elevated cobalt and/or chromium concentrations in their blood, serum, plasma, and/or urine, suggesting that these systemic symptoms may be linked to metal toxicity caused by excessive implant wear [13]. It is interesting to highlight that Posada et al. [13] observed how cobalt–chromium (CoCr) particles were capable of exerting a deleterious effect on U937 cells (a human leukemic monocyte lymphoma cell line). The authors demonstrated that exposure to the particles released from CoCr prostheses could induce a significant increase in cellular apoptosis and the overexpression of proteins related to inflammation processes, such as nitric oxide synthase 2 (NOS2) and bradyzoite-specific BAG1. Implementing biocompatible surface modifications on CoCr prostheses functionalized with GO and HA to mitigate wear–corrosion could effectively minimize debris release.

Previous extensive studies by the authors on the uniformity and coverage of GO functionalized with hyaluronic acid were analyzed using Fourier-transform infrared spectroscopy (FTIR) and X-ray photoelectron spectroscopy (XPS) [21], and Raman spectroscopy and electrochemical impedance spectroscopy (EIS) [22], respectively. The formation of a protective nanocoating based on the anchoring of GO onto the CoCr surface acted as an almost impermeable barrier due to the sp^2^ graphene network, which securely adhered to the metal surface. The XPS results revealed that three possible reactions ensure the strong chemical bonding between silanized CoCr surfaces and GO: the primary amines of the silane and epoxy groups of GO, the free –OH groups in the silane and carboxyl groups in GO, and the silane primary amines and –OH from the carboxyl groups of GO. Moreover, the interfacial bonding of GO to the metal surface was further reinforced by the subsequent immobilization of HA on the GO.

The authors have found that the presence of the GO/HA nanocoating exerts a significant lubricating effect in the tribocorrosion processes of the CoCr surface, reducing the wear track depth by 33% [22].

**Figure 1:**
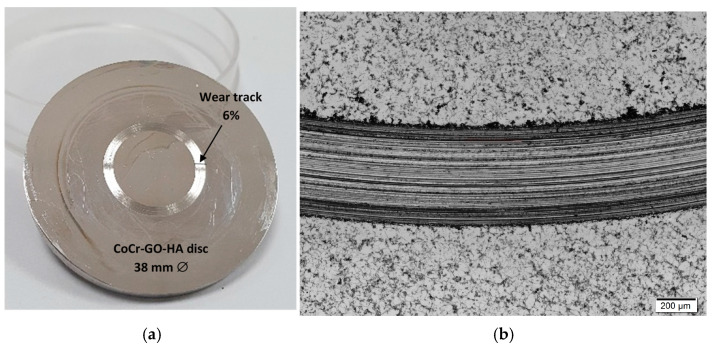
(**a**) Wear track on biofunctionalized CoCr surfaces after wear–corrosion tests at a load 5 N, 120 rpm at 30,000 m of sliding; (**b**) image taken from the optical microscope as an example to illustrate the coating’s uniformity of biofunctionalized CoCr surfaces after wear–corrosion tests, at a load 5 N, with alumina ball as counterpart, and 500 m of sliding. Wear–corrosion tests were performed in HA 3 g/L aqueous solution.

Figure 1 shows, as an example, the biofunctionalized CoCr surface after the wear–corrosion test. It has been observed that the estimated area of the worn surface represents 6% of the total tested surface. While GO-HA nanolayers can act as protective and lubricating barriers against physiological fluids when deposited on CoCr surfaces, they fail to fully cover the surface when subjected to wear–corrosion processes. The detached particles are either released into surrounding areas or transferred to worn regions, becoming incorporated into the surface. Nevertheless, the worn surface may continue to generate metallic debris in areas exposed to wear–corrosion [9,10,11,12,13,14,15,16]. The tested specimen partially reproduces an in vivo scenario, where wear–corrosion occurs in the sliding zones of a joint replacement prosthesis from the implantation, resulting in the coexistence of worn and unworn areas on the prosthesis surface.

**Figure 2:**
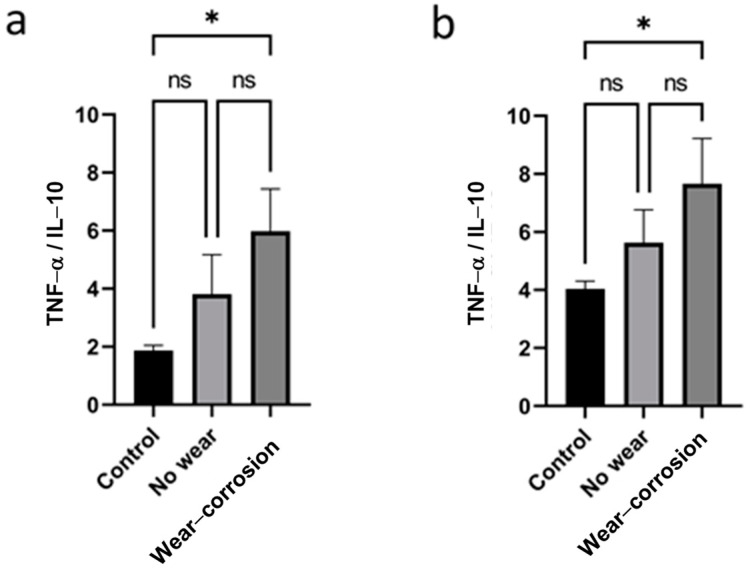
TNF-α/IL-10 values of J774A.1 macrophages cultured during (**a**) 72 h and (**b**) 96 h, on control (without material), on no wear–corrosion, and on wear–corrosion of CoCr-GO-HA. *p*-value < 0.05 (*); ns: non-significant differences.

The surfaces of these disks (see Figure 1), exhibiting both worn and unworn regions, have been subjected to macrophage cultures. In Figure 1b, an image from the optical microscope has been included as an example to illustrate the coating’s uniformity and coverage, as well as the disruption caused by the wear track from wear–corrosion tests carried out with a load of 5 N, with an alumina ball as a counterpart, and 500 m of sliding. Figure 2 depicts the average TNF-α/IL-10 ratio values after 72 h—Figure 2a—and 96 h—Figure 2b—of exposure in macrophage cultures, both before and after the wear–corrosion test. A control assay (without metallic material) was also included. As shown in Figure 2, the wear–corrosion of the CoCr-GO-HA disk produced an increase in the inflammatory response at both 72 and 96 h of material exposure concerning no-wear CoCr-GO-HA surfaces, but the differences were not significant. Only significant differences in the inflammatory response were observed between the worn CoCr-GO-HA surfaces and the control assay at both exposures—72 h and 96 h (Figure 2). On the other hand, the pro-inflammatory response of macrophages to worn CoCr surfaces decreased upon the covalent immobilization of HA and graphene oxide compared to the exacerbated pro-inflammatory response caused by abraded bare CoCr surfaces, previously reported by our group [24]. The authors reported TNF-α/IL-10 values after 72 h of 1.6, 27.34, and 82.76 for control, no wear, and with wear CoCr, respectively.

These results allow us to conclude that covalent modification with HA and GO on the CoCr surfaces significantly reduced the inflammatory response of the macrophages and seemed to be a good alternative to obtain a material surface with better cellular behavior, as inflammation was attenuated even in wear–corrosion conditions.

### 3.2. In Vivo Tests

#### 3.2.1. Debris Characterization

It would be of interest to analyze whether the proposed biofunctionalization of CoCr after wear–corrosion tests can induce a biological response to metal wear debris. So, debris from the corrosive test medium used in the wear–corrosion tests was collected after 30,000 m of sliding. The results from this investigation show that the semi-quantitative analysis of the debris performed by ICP-OES was 4 mg/L Co, 4.4 mg/L Cr, and 1.0 mg/L Si. The elements found in higher concentrations and practically in the same proportion are Co and Cr, both originating, especially, from the base material. The Si element originated from the APTES compound used as an intermediate for the covalent bonding of GO to the CoCr surface [21].

During revision surgeries or postmortem examinations, discolored tissue were often observed around CoCr implants, and some patients experienced unexplained pain that may have been related to the tissue damage caused by wear from MoM hip replacements [34]. Elevated levels of cobalt and chromium ions have been detected in peripheral blood and hip synovial fluid following CoCr alloy metal-on-metal (MoM) hip replacements, raising concerns about the toxicity and biological effects of these ions, both locally and systemically [35,36]. Cobalt corrodes more rapidly than chromium under physiological conditions [37]. Additionally, unlike chromium, cobalt ions tend to remain more mobile, as indicated by the higher levels detected in blood, enabling them to reach distant organs [38].

Once debris was centrifuged and the wear particles were extracted, as described in Section 2.1.4, the wear particles were dispersed in a 0.9% NaCl solution to be injected intra-articularly into the rats (Table 1).

The particles collected from the wear–corrosion process of the CoCr surfaces were previously characterized by the authors in terms of their morphology and chemical composition [39]. The analysis revealed that the particles were primarily composed of Cr, Co, Mo, P, C and O, originating from the surface oxide film. The particles were distinguished by an irregular morphology in the form of detached fragments of the oxide layer.

**Figure 3:**
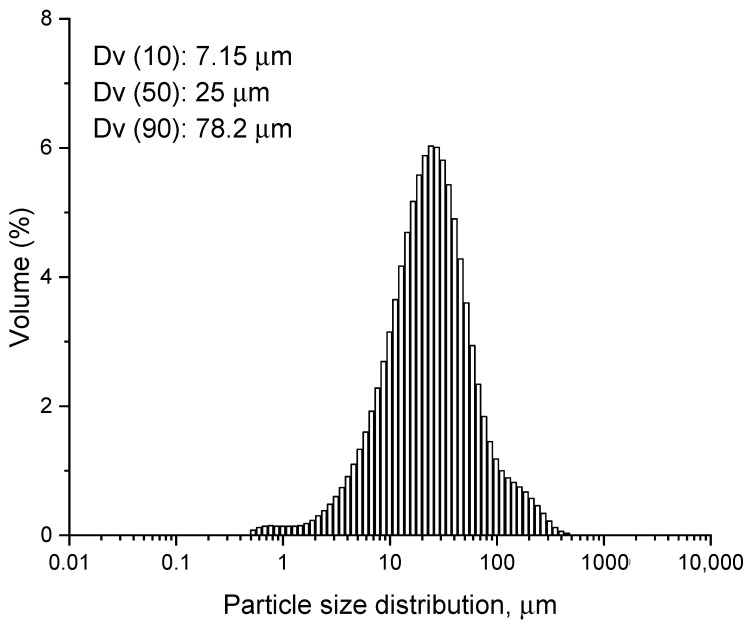
Distribution of the particle size after wear–corrosion tests.

The wear particle size distribution is illustrated in Figure 3. The Dv10, Dv50, and Dv90 percentiles of the particle size are 7.15 μm, 25 μm, and 78.2 μm, respectively. Only a small percentage of particle volume falls within the submicron range, specifically below 1 μm, likely due to the filtering method used, with a pore size of 0.10 μm. This suggests that the volume of material filtered as nano-particles is marginal, while a substantial portion is collected as larger particles.

The release of metallic debris in metal joint replacements primarily occurs in the form of micro-and nano-particles, ions of varying valences, and oxides composed of cobalt and chromium. The cobalt chromium alloy particles detected in MoM joint replacements were typically found in the nanometer range [40], while larger particles formed at fixation surfaces. Most wear particles can interact with the immune system and be engulfed by macrophages. Nanoscale and microscale wear particles interact differently with immune cells, leading to distinct biological responses. The smaller size of nano-particles allows them to adsorb more proteins, making them more recognizable to immune cells and often triggering a stronger immune response. Nano-particles are also taken up by cells through endocytosis or passive diffusion, allowing them to enter the cell more easily and activate inflammatory pathways, which can lead to the release of cytokines and potentially prolonged inflammation. Particles within the critical size range (~0.2–0.8 μm) [41] can activate macrophages and osteocytes, triggering a pro-inflammatory response and osteoclastogenesis, and contributing to aseptic loosening when produced in large quantities. Nevertheless, bad acetabular positioning, which leads to edge loading, promoted severe wear cases and larger micrometer-sized particles could also be found [42], potentially linked to the development of pseudo-tumors. Microparticles are typically taken up by phagocytosis, a process limited to specific immune cells like macrophages. This limits their intracellular interactions and generally results in a less intense immune response. According to scientific literature, it is well-established that macrophages cannot phagocytize particles larger than 25 μm due to their inability to penetrate cellular membranes. Due to their larger size, microparticles are more quickly recognized and cleared by immune cells, leading to a more localized response. The main material loss from the implant in the form of microparticles usually remains in the synovial cavity. On the other hand, nano-particles are more prone to be removed from the articular cavity and be transported to other parts of the body with different potential effects.

While these substances are known to alter biological processes, their direct effects on specific tissue types remain poorly understood. This knowledge gap may partly result from the use of diverse research methodologies, leading to inconsistent findings. In their review, Bijukumar et al. [15], highlight the need for new and more appropriate in vitro methodologies to better study the cellular responses and toxicity caused by wear debris in vivo from joint replacements.

In a previous similar research, the authors studied the biological implications caused by particles that were intraperitoneally injected [26]. This research represents an advanced step in the study of biological alterations caused by biofunctionalizing CoCr with graphene and hyaluronic acid by doing so in the rat’s patellar region in an attempt to resemble the debris locally produced during friction in replaced joint prostheses.

#### 3.2.2. Intra-Articular Injection of Particles from Wear–Corrosion Tests

The content of metal wear particles found in patients with joint replacements is highly variable, depending on factors such as the individual patient response, the implant design, the patient activity level, and the alignment precision during surgery. For instance, the misalignment of the implant can increase the rate of wear and lead to higher levels of metal debris. In cases of severe metallosis or adverse local tissue reactions, concentrations can reach several hundred micrograms per gram, indicating extensive metal release and accumulation. Lohmann et al. examined twenty-eight total hip arthroplasty implant retrievals performed due to pain and/or osteolysis, along with one patient who experienced recurrent dislocations. Intraoperatively, periprosthetic metallosis was observed in twelve cases, while the formation of a bursa (pseudotumor) was noted in thirteen. The study found that the metal content (cobalt, chromium, and nickel) in the periprosthetic tissue ranged from 1.4 to 4604.0 μg/g of dried tissue [43]. Other research conducted by Betts et al. measured the levels of cobalt, chromium, nickel, and molybdenum in periarticular tissue from 22 individuals who underwent revision surgery. They found that the total tissue content of these four elements ranged from 2.7 to 250 micrograms of metal per gram of dried tissue (mean: 39 micrograms/g). However, the metal content varied significantly among the individual cases [44].

In this work, the total mass of the particles generated during the wear–corrosion processes of biofunctionalized CoCr surfaces injected via intra-articular injection in Wistar rats varied from 18.9 to 70.5 µg (in 30 µL), taking into account the smaller size and lower weight of a rat of 250 g. An in vivo model with the intra-articular injection of particles would analyze the possible movement of subparticles and microparticles from the intra-articular cavity. Following injection, none of the rats showed symptoms of limping either immediately after the intra-articular injection or throughout the experimentation.

**Figure 4:**
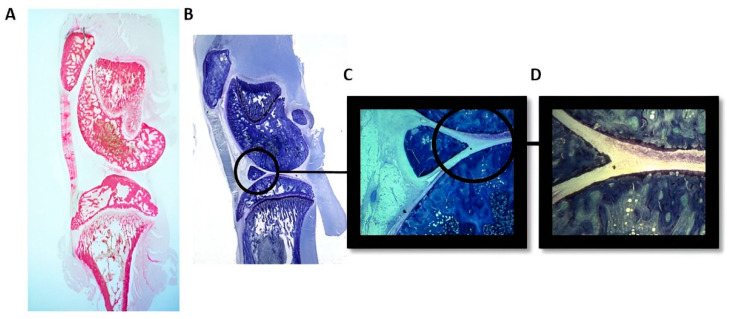
Histology images of the knee joint: (**A**) control stained with hematoxylin–eosin (0.8× magnification in binocular magnifying glass); (**B**) particles after 30 days of intra-articular injection stained with toluidine blue (0.6× magnification in binocular magnifying glass); (**C**) 4× magnification in binocular magnifying glass; and (**D**) 25× microscope magnification.

Figure 4A shows a global image with hematoxylin–eosin staining, showing the knee joint assembly where particles with different concentrations were injected. It was a parasagittal section of the knee joint, where the tibial bone was seen at the bottom and the femoral condyle at the top, with the patella in front of it. The patellar tendon extended downward from the patella, inserting into the anterior part of the tibia.

Figure 4B shows, according to toluidine blue staining, the overall image of a parasagittal section of the knee joint assembly in an animal injected with wear particles. Among the different injected concentrations, particles were only detected in the joint at the highest concentration of 2.35 mg/mL—Figure 4B–D. In images Figure 4B,C, particles can be observed within circles one month after the experiment, which coincides with the characteristics of the injected particles.

##### Hematological Results

The groups 0 (control) and 1 (hyaluronic) were compared using a non-parametric test, the Mann–Whitney U test, to determine if merging the two groups is possible. No significant differences were found between the distributions of both of the groups for any considered variables. Therefore, both of the groups were regrouped for further analysis.

Table 2 shows that the hematological values were average, with no statistically significant differences between the values of the control group and the rest of the injected groups. Neither the red cell series, white cell series, liver enzymes (Aspartate aminotransferase (AST) and alanine aminotransferase (ALT)), nor the coagulation system (platelet (PLT) counts, prothrombin time (PT), and thrombin time (TT)) were altered.

**Figure 5:**
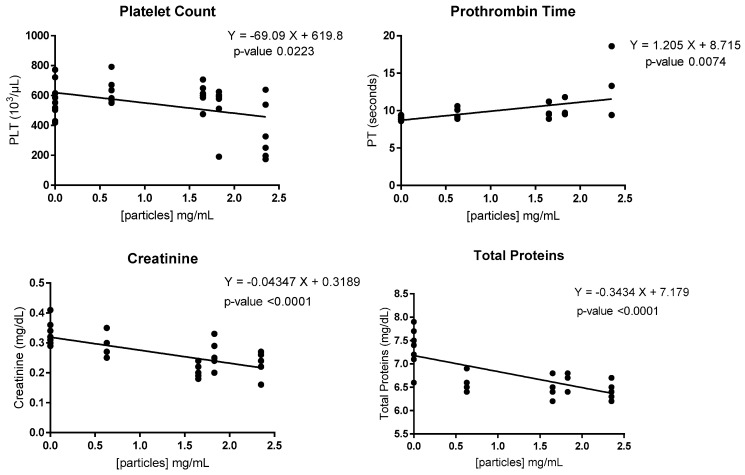
Graphical representation of the linear relationship between statistically significant variables observed with Pearson’s relationship and its equation.

**Figure 6:**
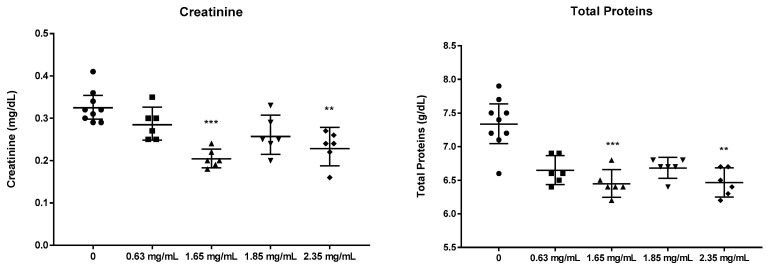
Comparative analysis of creatinine (**left**) and total proteins (**right**) at increasing particle concentrations. Note that only parameters with statistically significant differences after applying the non-parametric Kruskal–Wallis test and subsequent correction with the Dunn test have been represented (** *p*-value < 0.01, *** *p*-value < 0.001).

Figure 5 shows the linear relationship between the significant variables observed with Pearson’s relationship and its equation. Although Pearson’s correlation showed statistically significant relationships between particle concentration and the variables’ platelet (PLT) counts, the prothrombin time (PT), creatinine (Crea), and the total protein values (Figure 5), the subsequent comparative analysis by groups using the non-parametric Kruskal–Wallis test, and subsequent correction with the Dunn test, statistically significant results were only obtained for the total protein and creatinine values (Figure 6).

Although some authors have previously used the intra-articular route in the rat knee for their research [45]; it has not been found in recent studies in the literature on the biocompatibility or toxicity of graphene and its derivatives via the intra-articular route in experimental animals. The most commonly used administration routes in rodents are inhalation, oral, intravenous, or intraperitoneal routes [26]. The particles do not cause short-term injuries, considering the synovial membrane participates in the intra-articular immune response [46].

The results from the animal model demonstrate an absence of inflammatory response, aligning with our in vitro findings. It is important to note that the materials studied under the experimental conditions in this study differ between in vitro and in vivo: in vitro tests are focused on surfaces, while in vivo tests evaluate the hematological response to particles generated from wear–corrosion tests. Nonetheless, both approaches yield a similar trend in inflammatory outcomes. In vitro results combined with those described in the animal model suggest that using graphene oxide functionalized with hyaluronic acid on CoCr alloys could substantially improve the quality of joint prostheses and their potential impact on patient health.

The results of the in vivo model in CoCr joint prosthesis can be compared with the work of Eichenbaum et al. [9], Matusiewicz et al. [11] or Bijukumar et al. [15], in which they observed systemic and local alterations induced by the effect of chromium and cobalt metal particles. Thus, in their works they determined local alterations such as inflammation, oxidative stress, granuloma or pseudotumor formation, adverse tissue reactions, macrophage activation, endothelial cell activation and osteolysis, which can contribute to chronic inflammation and compromise the longevity of the implants. Additionally, cobalt and chromium ions can lead to systemic alterations proven such as cardiomyopathy, hypothyroidism, neurotoxicity, carcinogenesis, genotoxicity, renal and hepatic toxicity, and immunological alterations. In addition, at the cellular level, they also observed that metal molecules could induce chronic inflammation and oxidative stress.

Regarding the animal model in this work, the changes found in the altered blood parameters generally do not indicate tissue or organ damage (Table 2 and Figure 5 and Figure 6). The only results with statistically significant differences were creatinine and total proteins (significantly lower in group 6 vs. control group, in Figure 6). Both the parameters were related to renal function and suggested a possible phenomenon of glomerular hyperfiltration, a phenomenon that can occur due to different clinical conditions, including renal disease [47]. Patlolla et al. [48], after the oral administration of much higher doses (20 and 40 mg/kg) than those used in the present study, observed the oxidative stress and tubular dysfunction of the kidney in Sprague Dawley Rats. Some authors suggest that the kidney is one of the main routes of the excretion of nanomaterials from the body [49], which could help explain this phenomenon. However, the results show a statistically significant difference, which does not indicate pathological change. In absolute terms, the observed variations do not suggest renal damage. In the 2022 study by Moreno-Gómez-Toledano et al. [50], the administration of streptozotocin led to a significant increase in serum creatinine, commonly associated with renal damage. Additionally, the plasma albumin levels dropped below 50%, indicating a nephrotic range where the liver could not compensate for urinary protein loss.

Conversely, our current animal model showed only slight reductions in creatinine and protein, within non-pathological ranges for the mouse’s pathophysiological context. Therefore, the results suggest a minor hyperfiltration process, which is logical given that nano-particles and microparticles are primarily excreted via the urinary route.

In this work, wear debris was proven to be nontoxic in rats, showing that wear debris is likely metabolized in the kidney. Co and Cr ions and other heavy metals are the primary binding substrates for glutathione, making them water-soluble for kidney excretion [51,52]. Nevertheless, the mechanism of the excretion of these substances is still under study [53]. Comprehensive studies over an extended testing period will be required in the future to clarify this matter.

Huixia Wu et al.’s study [54] shows good blood and tissue compatibility in mice ten days after intravenous administration of GO-HA in the tail vein. They found no blood alterations in the red, white, and platelet series nor alterations in liver or kidney function. The exposure time to the treatment was notably shorter (10 days) than ours (30 days), and the administration route was also different.

On the other hand, according to Mao et al. [55], after intra-tracheal instillation in mice, the prolonged graphene in the lung only caused transient pulmonary effects, as the acute symptoms reversed over time despite the maintenance of chronic graphene exposure in the lungs.

The work by Alves da Silva et al. [56], when reviewing the current literature of the last ten years, shows the potential toxic effect from exposure to different forms of graphene in a dose-dependent manner, generally, but not exclusively, in respiratory exposure (given that this is the most common exposure in humans) [57]. The first study on controlled exposure to inhaled graphene oxide in humans, consisting of graphene oxide nanosheets at a concentration of 200 μg/m^3^ inhaled for 2 h by 14 young, healthy volunteers in repeated visits, showed no adverse effects in their lung function, blood pressure, or blood parameters, including the coagulation system.

Finally, although the interaction between graphene and various cells, tissues, and organs is still to be fully elucidated, a differentiation effect on osteoblasts and antibacterial capacity has been confirmed, which supports graphene as a material for application in engineering in bone and cartilage tissue [58].

## 4. Conclusions

The surface modification of the implant with GO and HA did not have any detrimental effects in either the in vitro or animal models under the experimental variables of this study.

The in vitro inflammatory response of the macrophages to the covalent modification with HA and GO on the CoCr surfaces was minimally altered compared to the control. However, following wear–corrosion tests, although the inflammatory response increased, the change was less pronounced than that induced by the worn CoCr surfaces without modification. The inflammatory response remained attenuated, even after the wear–corrosion process.

The intra-articular injection technique proposed here in the experimental studies demonstrates its validity and essential role for broader and future research endeavors. Particle remnants were found at the maximum concentration that was tested in the joints, but without provoking inflammatory signs.

The hematological analysis did not reveal any pathological damage to the tissues or organs. The only parameters that showed statistically significant differences were the creatinine and total proteins, both of which are related to renal function. However, the changes were mild and did not affect the current health status of the animal, let alone reach the nephrotic range.

The results have shown the potential benefits of using graphene oxide and hyaluronic acid in intra-articular prostheses, which could improve the quality of life for numerous patients.

## Figures and Tables

**Table 1:** Experimental design.

Group	Number of Rats
0—Control	6
1—Hyaluronic acid	6
2—Reduced graphene oxide	6
3—Wear particles (0.63 mg/mL)	6
4—Wear particles (1.65 mg/mL)	6
5—Wear particles (1.83 mg/mL)	6
6—Wear particles (2.35 mg/mL)	6

**Table 2:** Hematological values.

Blood Parameters	Control (Group 0)	Hyaluronic (Group 1)	Graphene (Group 2)	Wear Particles			
				[0.63 mg/mL] (Group 3)	[1.65 mg/mL] (Group 4)	[1.83 mg/mL] (Group 5)	[2.35 mg/mL] (Group 6)
RBC (10^6^/µL)	8.13 ± 0.37	8.39 ± 0.52	8.45 ± 0.76	8.39 ± 0.44	8.27 ± 0.28	8.24 ± 0.38	8.20 ± 0.41
Hgb (g/dL)	15.07 ± 0.49	15.33 ± 0.80	14.75 ± 0.76	14.85 ± 0.53	14.93 ± 0.73	14.58 ± 0.67	14.98 ± 0.42
Hct (%)	41.9 ± 2.47	43.35 ± 1.96	42.60 ± 4.19	42.63 ± 1.95	42.05 ± 1.67	41.70 ± 3.10	41.83 ± 1.36
MCV (fL)	51.5 ± 0.94	51.73 ± 1.47	50.38 ± 1.21	50.68 ± 0.38	50.78 ± 0.97	50.48 ± 1.52	51.00 ± 1.27
MCHC (g/dL)	35.63 ± 0.17	35.30 ± 0.30	35.58 ± 0.34	35.53 ± 0.57	35.33 ± 0.33	35.40 ± 0.40	35.08 ± 0.27
WBC (10^3^/mm^3^)	5.96 ± 0.69	6.83 ± 1.19	7.05 ± 1.99	7.44 ± 1.16	7.18 ± 3.45	6.56 ± 1.86	6.75 ± 2.14
PLT (10^3^/µL)	522.6 ± 67.5	591.83 ± 136.26	475.67 ± 194.95	633.17 ± 89.82	605.00 ± 77.05	517.00 ± 164.73	354.00 ± 192.05
PT (sg)	9.25 ± 0.15	8.94 ± 0.27	28.17 ± 44.94	9.51 ± 0.67	9.74 ± 0.86	10.06 ± 0.97	13.76 ± 4.61
TT (sg)	41.15 ± 11.15	40.16 ± 12.32	45.20 ± 14.61	44.61 ± 8.21	57.82 ± 12.77	51.38 ± 22.69	52.66 ± 30.30
F (mg/dL)	150.15 ± 28.25	145.5 ± 35.98	109.73 ± 57.48	148.66 ± 29.79	147.47 ± 16.81	144.72 ± 13.40	176.20 ± 15.55
Crea (mg/dL)	0.32 ± 0.02	0.33 ± 0.05	0.23 ± 0.02	0.28 ± 0.03	0.20 ± 0.02	0.26 ± 0.04	0.23 ± 0.03
Total proteins (g/dL)	7.13 ± 0.45	7.45 ± 0.26	6.74 ± 0.37	6.65 ± 0.20	6.45 ± 0.19	6.68 ± 0.14	6.46 ± 0.20
AST (UI/L)	84.0 ± 2.94	77.17 ± 17.84	78.43 ± 17.96	79.83 ± 10.00	76.66 ± 11.44	84.00 ± 11.45	77.83 ± 17.78
ALT (UI/L)	37.0 ± 4.9	37.67 ± 10.65	35.00 ± 7.12	34.00 ± 5.86	36.50 ± 8.01	36.00 ± 2.44	35.00 ± 3.16
LDH (UI/L)	507.67 ± 155.38	326.33 ± 62.73	395.29 ± 160.23	434.00 ± 166.2	392.00 ± 90.95	434.20 ± 113.80	290.30 ± 79.06
CHO (mg/mL)	103.33 ± 15.80	103.50 ± 21.35	100.57 ± 19.61	100.83 ± 9.78	99.66 ± 7.17	92.00 ± 11.62	97.33 ± 10.01
TG (mg/mL)	156.0 ± 29.22	183.66 ± 68.58	148.00 ± 36.56	165.50 ± 33.44	189.50 ± 45.05	198.30 ± 37.60	180.70 ± 63.70

ALT: alanine aminotransferase; AST: aspartate aminotransferase; CHO: cholesterol; crea: creatine; F: fibrinogen; Hct: hematocrit; Hgb: hemoglobin; LDH: lactate dehydrogenase; MCHC: mean corpuscular hemoglobin concentration; MCV: mean corpuscular volume; PLT: platelet count; PT: prothrombin time; RBC: red blood cells; TG: triglyceride; TT: thrombin time; WBC: white blood cells. Each value represents the mean ± SD.

## Data Availability

Data have been deposited in the institutional repository of the CSIC (Digital CSIC) http://hdl.handle.net/10261/367517 (accessed on 11 September 2024).
